# Rapid virtual fractional flow reserve using 3D computational fluid dynamics

**DOI:** 10.1093/ehjdh/ztad028

**Published:** 2023-04-21

**Authors:** Thomas Newman, Raunak Borker, Louise Aubiniere-Robb, Justin Hendrickson, Dipankar Choudhury, Ian Halliday, John Fenner, Andrew Narracott, D Rodney Hose, Rebecca Gosling, Julian P Gunn, Paul D Morris

**Affiliations:** Department of Infection, Immunity and Cardiovascular Disease, The Medical School, University of Sheffield, Beech Hill Road, Sheffield, S10 2RX, UK; Department of Cardiology, Sheffield Teaching Hospitals NHS Foundation Trust, Chesterman Wing, Northern General Hospital, Herries Road, Sheffield, S5 7AU, UK; Ansys Incorporated, Canonsburg, PA 15317, USA; Department of Infection, Immunity and Cardiovascular Disease, The Medical School, University of Sheffield, Beech Hill Road, Sheffield, S10 2RX, UK; Ansys Incorporated, Canonsburg, PA 15317, USA; Ansys Incorporated, Canonsburg, PA 15317, USA; Department of Infection, Immunity and Cardiovascular Disease, The Medical School, University of Sheffield, Beech Hill Road, Sheffield, S10 2RX, UK; Insigneo Institute for In Silico Medicine, Pam Liversidge Building, The University of Sheffield, Broad Lane, Sheffield, S1 3JD, UK; Department of Infection, Immunity and Cardiovascular Disease, The Medical School, University of Sheffield, Beech Hill Road, Sheffield, S10 2RX, UK; Insigneo Institute for In Silico Medicine, Pam Liversidge Building, The University of Sheffield, Broad Lane, Sheffield, S1 3JD, UK; Department of Infection, Immunity and Cardiovascular Disease, The Medical School, University of Sheffield, Beech Hill Road, Sheffield, S10 2RX, UK; Insigneo Institute for In Silico Medicine, Pam Liversidge Building, The University of Sheffield, Broad Lane, Sheffield, S1 3JD, UK; Department of Infection, Immunity and Cardiovascular Disease, The Medical School, University of Sheffield, Beech Hill Road, Sheffield, S10 2RX, UK; Insigneo Institute for In Silico Medicine, Pam Liversidge Building, The University of Sheffield, Broad Lane, Sheffield, S1 3JD, UK; Department of Infection, Immunity and Cardiovascular Disease, The Medical School, University of Sheffield, Beech Hill Road, Sheffield, S10 2RX, UK; Department of Cardiology, Sheffield Teaching Hospitals NHS Foundation Trust, Chesterman Wing, Northern General Hospital, Herries Road, Sheffield, S5 7AU, UK; Insigneo Institute for In Silico Medicine, Pam Liversidge Building, The University of Sheffield, Broad Lane, Sheffield, S1 3JD, UK; Department of Infection, Immunity and Cardiovascular Disease, The Medical School, University of Sheffield, Beech Hill Road, Sheffield, S10 2RX, UK; Department of Cardiology, Sheffield Teaching Hospitals NHS Foundation Trust, Chesterman Wing, Northern General Hospital, Herries Road, Sheffield, S5 7AU, UK; Insigneo Institute for In Silico Medicine, Pam Liversidge Building, The University of Sheffield, Broad Lane, Sheffield, S1 3JD, UK; Department of Infection, Immunity and Cardiovascular Disease, The Medical School, University of Sheffield, Beech Hill Road, Sheffield, S10 2RX, UK; Department of Cardiology, Sheffield Teaching Hospitals NHS Foundation Trust, Chesterman Wing, Northern General Hospital, Herries Road, Sheffield, S5 7AU, UK; Insigneo Institute for In Silico Medicine, Pam Liversidge Building, The University of Sheffield, Broad Lane, Sheffield, S1 3JD, UK

**Keywords:** Fractional flow reserve, Computational fluid dynamics, Computer modelling

## Abstract

**Aims:**

Over the last ten years, virtual Fractional Flow Reserve (vFFR) has improved the utility of Fractional Flow Reserve (FFR), a globally recommended assessment to guide coronary interventions. Although the speed of vFFR computation has accelerated, techniques utilising full 3D computational fluid dynamics (CFD) solutions rather than simplified analytical solutions still require significant time to compute.

**Methods and results:**

This study investigated the speed, accuracy and cost of a novel 3D-CFD software method based upon a graphic processing unit (GPU) computation, compared with the existing fastest central processing unit (CPU)-based 3D-CFD technique, on 40 angiographic cases. The novel GPU simulation was significantly faster than the CPU method (median 31.7 s (Interquartile Range (IQR) 24.0–44.4s) vs. 607.5 s (490–964 s), *P* < 0.0001). The novel GPU technique was 99.6% (IQR 99.3–99.9) accurate relative to the CPU method. The initial cost of the GPU hardware was greater than the CPU (£4080 vs. £2876), but the median energy consumption per case was significantly less using the GPU method (8.44 (6.80–13.39) Wh vs. 2.60 (2.16–3.12) Wh, *P* < 0.0001).

**Conclusion:**

This study demonstrates that vFFR can be computed using 3D-CFD with up to 28-fold acceleration than previous techniques with no clinically significant sacrifice in accuracy.

## Graphical Abstract

**Graphical Abstract:**
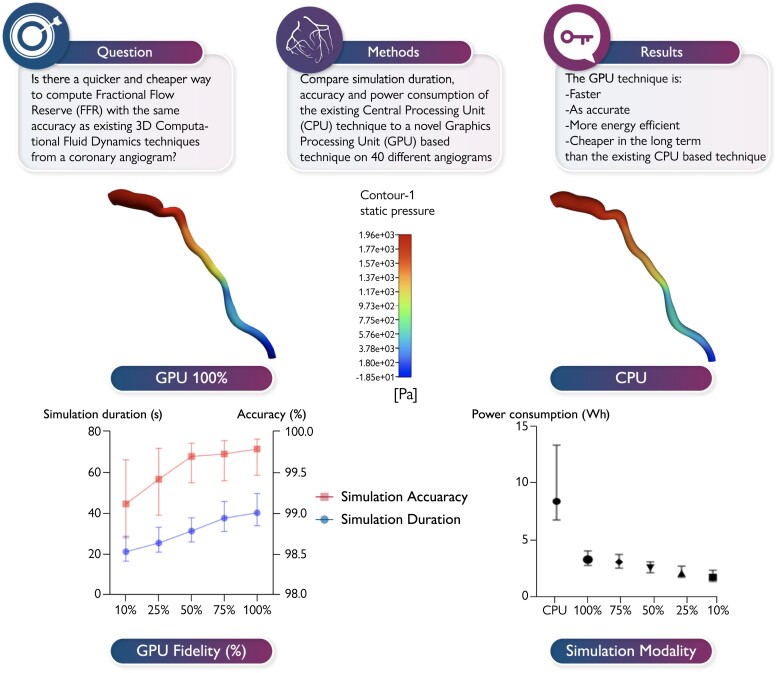

## Introduction

Fractional flow reserve (FFR) is the gold-standard assessment of physiological lesion significance in the cardiac catheter laboratory and is useful in guiding percutaneous coronary intervention. It has a class 1A indication in the major international clinical guidelines being shown to reduce mortality, myocardial infarction, healthcare costs, and stent deployments.^1,2^ Fractional flow reserve is the ratio of the time averaged pressure distal to a coronary stenosis to that proximal. Fractional flow reserve is a semiquantitative index used as a surrogate for the percentage flow limitation due to epicardial coronary artery disease. Fractional flow reserve, however, remains underused due to the requirement for an expensive, single-use pressure wire, and the induction of hyperaemia.^3,4^ In well-resourced settings, FFR use is 10–20%, but in less well-resourced health services, the use of pressure wires is close to zero. Methods have therefore been developed to derive FFR from the angiogram, without the need for a pressure wire or inducing hyperaemia. Since the original method described by Morris *et al*. in 2013, several systems have become approved for clinical use, typically designated as angiography-derived or ‘virtual’ FFR (vFFR).^5,6^ Virtual fractional flow reserve predicts invasive FFR with a 95% confidence interval of around FFR ±0.12, predicts physiological significance (FFR ≤0.80) with ∼85–95% accuracy, and has recently been shown to result in improved clinical outcomes compared to traditional angiographic guidance.^6^

Methods for deriving vFFR from the angiogram require two 2-Dimensional (2-D) angiographic projections to be reconstructed into a 3D model of the diseased artery. Flow computation based upon the laws of fluid dynamics is used to calculate the translesional pressure drop, from which the vFFR is calculated as the ratio of the distal and proximal pressures. Three-dimensional computational fluid dynamics (CFD) is widely regarded as the gold-standard mathematical solution relying on the Navier–Stokes equations that describe the dynamics of incompressible, viscous fluids.^7,8^ This approach, however, requires substantial computer processing time and power, solving in several minutes or even hours. For this reason, many commercial vFFR methods apply simpler, analytical solutions based upon the laws of Bernoulli and/or Poiseuille for instantaneous calculation. These are more attractive for providing immediate ‘on-table’ results for supporting clinical decision making, but the use of a simplified solution could neglect components of complex translesional physiology that may adversely affect the accuracy of vFFR.

In 2013, the original vFFR method using CFD took >24 h to compute, based upon a fully transient, 3D-zero-dimensional coupled model.^5^ In 2017, an accelerated method was developed that retained the benefits and accuracy of full 3D-CFD, but enabled 500-fold acceleration, with results in 3 min.^9^ The aim of this study was to develop and validate an even faster 3D-CFD method capable of accurate vFFR results in <30 s. We compared the new method against the current gold-standard 3D-CFD method.

## Methods

This study was performed at the University of Sheffield, UK and at Ansys Inc, Research and Development Department, Canonsburg, USA. The Sheffield team collected the clinical data and constructed the coronary models, and the Ansys team performed the CFD analyses. The study was approved by the regional institutional and ethics boards. All data underlying this study are included in the manuscript or the supplementary appendix.

### Clinical data collection

Anonymized imaging data were collected from 35 patients with coronary artery disease who underwent invasive angiography for chronic coronary syndromes at Sheffield Teaching Hospitals NHS Trust, UK. Patients were included if they had coronary disease with one or more diameter stenosis 30–90% by visual estimation as agreed by three experienced cardiologists. Anonymized data regarding comorbidities and demographics were also collected. Coronary angiography and measurement of invasive FFR were performed using standard techniques.^9,10^ Invasive FFR was performed based upon operator discretion for clinical assessment rather than for research purposes.

### Computing virtual fractional flow reserve

Coronary anatomy was reconstructed using the VIRTUheart™ (University of Sheffield, UK) model of coronary physiology. Digital Imaging and Communications in Medicine (DICOM) data from the coronary angiograms were reconstructed from paired images acquired ≥30° apart using an epipolar line transection method that corrects for between-acquisition movement of the patient and table and validated previously.^11,12^ A .stl computer file was generated that defined the 3D anatomy of the diseased coronary arterial lumen. This was used as the basis for CFD simulation. Both CFD methods compared in this study used paired steady-state simulations at 1 and 3 mL/s with a plug velocity profile and a uniform zero-pressure outlet. The results of this analysis were used to derive the linear (viscous) and quadratic (inertial) terms within a quadratic equation that characterized the relationship between pressure and flow for each case. The vFFR was then calculated using the pseudo-transient method described previously.^9^ This approach was not altered, but the technique of solving the CFD equations was, using either a novel graphics processing unit (GPU)-based approach that was compared to the reference method that was the central processing unit (CPU) method. Virtual fractional flow reserve was measured at the same point along the artery in both CPU and GPU methods. This was identical to the *in vivo* measured FFR if performed or from the position it would have been measured as determined by the senior clinical operator if it was not measured *in vivo*.

### Central processing unit reference method

Simulations were performed using parallel processing on four cores of an Intel Xeon 6136 Gold CPU (Intel Corporation, USA). Ansys Fluent™ (Ansys Inc., USA) was used as the benchmark CFD solver. It uses the well-established finite volume method to discretize the incompressible Navier–Stokes equations and solves them using a pressure based solver to steady-state flow.^13,14^ Ansys Fluent™ meshing was used to generate unstructured poly meshes with prism layers at the boundary. Meshes had between 1 and 2 million cells depending upon the geometry of the angiogram. For the size of meshes used here, reduction in simulation duration does not scale linearly with the number of processor cores greater than four, so no additional cores were used. The results from this approach represent the current gold-standard benchmark and are referred to as the CPU method.

### Graphics processing unit method

Ansys Discovery Explore™ was used as the experimental CFD solver. It uses the CFD methods described above as in Fluent™ but the meshes used to discretize the geometry are automatically generated in Discovery™. The time required for meshing was not measured or included for either CPU or GPU methods in this assessment although meshing duration tends to be faster in the integrated workflow of the GPU method. In this study, multiple mesh fidelities (resolutions) were tested, with mesh sizes ranging from 1 to 10 million cells. A higher fidelity implies that more cells (equivalent to pixels in images) are used to represent the geometry. Fidelity is varied using a slider control in the user interface from 1% to 100%, in which higher percentages reflect higher cell discretization. In this study, five levels of fidelity were tested; 10, 25, 50, 75, and 100%. Unlike the CPU method, Discovery Explore™ runs exclusively on a GPU. The performance of the Discovery Explore solver is not affected by the specification of the CPU on the computer. This novel method is referred to as the GPU method.

In this study, both CPU and GPU simulations were performed on the same computer equipped with an Nvidia RTX 6000 GPU paired with an Intel Xeon 6136 Gold CPU. The Nvidia RTX 6000 is a mid- to high specification GPU with a memory of 24GB. The Xeon 6136 Gold is also mid- to high specification with a total of 12 total cores and maximum frequency of 3.7 GHz although only four cores were utilized in this experiment as described above. These systems are both in common use in hospitals and research institutions.

### Accuracy and duration analysis

The primary outcome measures of this study were the speed and accuracy of the novel GPU method, compared with the CPU method. The time taken for each modality to complete CFD simulation and produce a vFFR result was recorded, in seconds. Accuracy was calculated as the percentage difference in the GPU-derived vFFR relative to the CPU-derived result given by


$$\left(1\;-\left(\frac{\left|\mathrm{vFF}R_{\mathrm{GPU}}\;-\;\mathrm{vFF}R_{\mathrm{CPU}}\right|}{\;\mathrm{vFF}R_{\mathrm{CPU}}}\right)\right)\times 100.$$

### Assessing the cost of computing the virtual fractional flow reserve

The cost of computing vFFR was assessed by two metrics: the initial acquisition cost of the hardware; and the energy required to run the simulation. The Intel Xeon 6136 Gold CPU draws an average power of 150 W to supply its 12 cores. Because only four cores were used in this study, and the power to core profile is not known, $P_{\mathrm{CPU}}$ = 50 W was used as a conservative estimate of average power consumption for four cores. The Nvidia RTX 6000 GPU has a maximum power consumption of 295 W that was used as the average power consumption.

### Statistical analysis

Analysis and data recording were performed with Microsoft Excel (Microsoft Corporation, Washington, USA) and GraphPad Prism 9.3.1 (GraphPad Software Inc.). Data were assessed for parametric distribution using the Shapiro–Wilk test. Parametric data were summarized with mean +/−95% confidence interval and non-parametric summarized with median +/− interquartile range (IQR) and results presented in the same way. When comparing results at different fidelities, each angiogram was analyzed at each fidelity, so results are paired. Paired non-parametric comparisons were tested using the Wilcoxon sign rank test. To assess accuracy of the GPU method at different fidelities Bland–Altman plots of the difference between the vFFR computed by the CPU method and the GPU method at different fidelities against the mean vFFR. Simple linear regression was used to explore the relationship between CPU vFFR and the accuracy of the GPU technique at different fidelities. GraphPad Prism’s inbuilt test to compare slopes was used equivalent to the analysis of covariance.^15^

## Results

Forty-two angiograms from 35 patients were obtained. Meshes of two vessels could not be created in the CPU method, but could be in the GPU method, and these were excluded from comparative analyses. Therefore, 40 angiograms from 34 patients underwent simulation by both the CPU and GPU methods and were included in the final analysis. The baseline patient and vessel characteristics are summarized in *Table 1*. Of the 40 ‘meshable’ coronary arteries, 22 were of the left anterior descending (LAD), seven left circumflex (LCx), eight right (RCA), two obtuse marginal (OM), and one left main stem (LMS). The median lesion stenosis was 60% (IQR 50–70%) and median invasively measured FFR 0.78 (0.69–0.86). Data regarding characteristics and comorbidities were missing for one patient with data regarding percentage stenosis and FFR missing for two angiograms.

**Table 1:** Characteristics of patients and vessels

Patient characteristics	Median (IQR)/frequency (%)
Patients	34
Age (years)	64 (57–69)
Female	9 (27.3)
Overweight or obese (BMI >25)	20 (60.6)
White ethnicity	31 (93.9)
Patient comorbidities	
Diabetes	7 (21.2)
Current smoker	6 (18.2)
Prior myocardial infarction	11 (33.3)
Hypertension	24 (72.7)
Hyperlipidaemia	25 (75.8)
Vessel characteristics	*n* = 40
LAD	22 (55%)
LCx	7 (17.5%)
RCA	8 (20%)
OM	2 (5%)
LMS	1 (2.5%)
Percentage stenosis	60 (50–70%)
FFR	0.78 (0.69–0.86)

*n* = 33 for all patient variables and *n* = 38 for percentage stenosis and FFR.

LAD, left anterior descending artery; LCx, left circumflex artery; RCA, right coronary artery; OM, obtuse marginal artery; LMS, left main stem.

### Simulation duration

**Figure 1:**
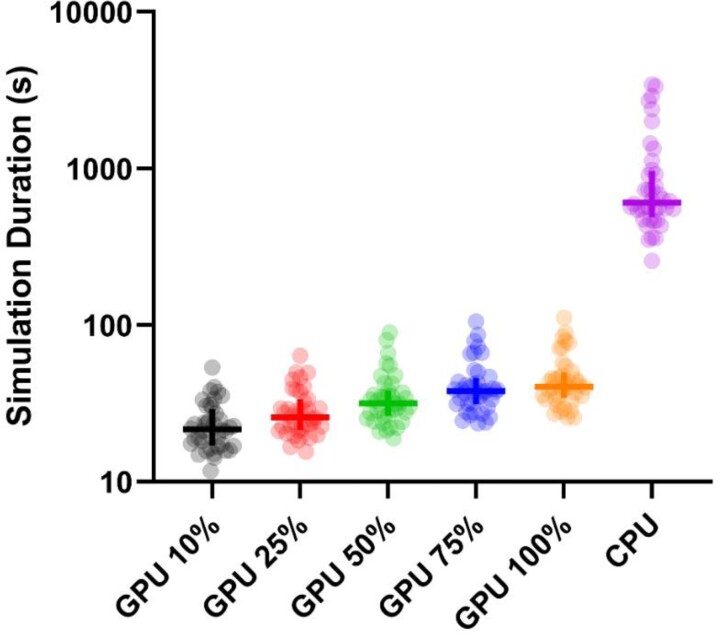
Simulation duration of different simulation methods. Dots represent each case simulated, horizontal bars the median value of that method, and vertical bars the interquartile range. Note logarithmic *y* axis.

The median CPU simulation duration was 607.5 (IQR 490–964)s, significantly longer than the median GPU simulation duration that, with all fidelities pooled, was 31.7 (24.0–44.4)s (*P* < 0.0001). Of the GPU fidelities, 10% fidelity produced the fastest results [21.5 (17.0–29.1)s], and 100% fidelity was the slowest [40.5 (34.2–49.9)s] (*Figure 1*). Each higher increment in fidelity led to a statistically significant increase in the simulation duration (*Table 2*). These differences were small but consistent with the stepwise increase in fidelity. The maximum difference in simulation duration on the same angiogram when fidelity was varied from 10% to 100% was 72.5 s (16.7 s at 10% to 89.3 s at 100%). Comparing the median of all angiogram results, the GPU method was 93.3% faster when comparing 100% fidelity with the CPU method and 96.5% faster when comparing the 10% fidelity method with CPU computation.

**Table 2:** Results of Wilcoxon sign rank pair testing comparing simulation duration using different computation methods

Comparison	Median of difference in simulation duration (s) [96.15% confidence interval]	*P* value
GPU 10% vs. GPU 25%	4.62 [4.11–5.92]	<0.0001
GPU 25% vs. GPU 50%	4.63 [4.12–5.68]	<0.0001
GPU 50% vs. GPU 75%	4.75 [3.97–5.93]	<0.0001
GPU 75% vs. GPU 100%	3.72 [2.81–4.40]	<0.0001
GPU 10% vs. GPU 100%	18.49 [16.17–21.11]	<0.0001
Median of all GPU vs. CPU	581 [517–703]	<0.0001

### Simulation accuracy

**Figure 2:**
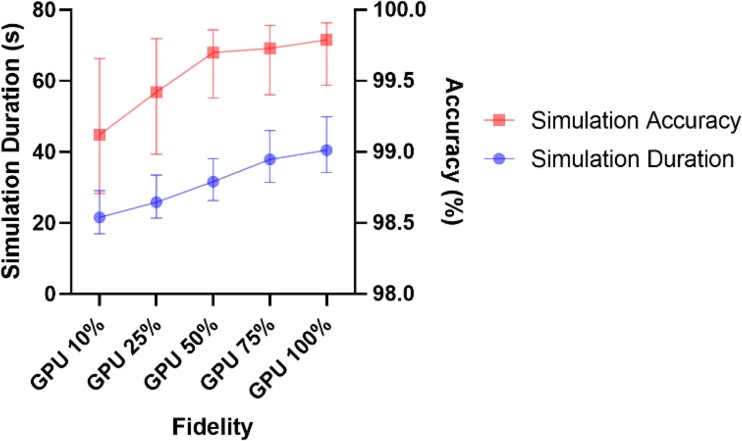
The impact of varying fidelity on simulation accuracy and simulation duration. Plots of median +/− IQR.

Accuracy of each fidelity of GPU simulation was compared against the CPU method (*Figure 2*). The lowest level of accuracy for any single simulation was 97.5% with a median accuracy of 99.6% (IQR 99.3–99.6%) when results were pooled across all fidelities. Ten per cent fidelity had the lowest median accuracy of 99.1% whilst 100% fidelity had accuracy of 99.8%. Each increase in fidelity produced a small but statistically significant gain in accuracy (*Table 3*). The effect of measuring vFFR at different points along the angiogram was also compared in four examples covering the range of vFFRs computed (see Supplementary material online, *Appendix section A.2*).

**Table 3:** Results of Wilcoxon sign rank pair test comparisons of simulation accuracy using different computation methods relative to the central processing unit method

Comparison	Median of difference in accuracy (%) [96.15% confidence interval]	*P* value
GPU 10% vs. GPU 25%	0.15 [0.10–0.23]	<0.0001
GPU 25% vs. GPU 50%	0.11 [0.07–0.20]	<0.0001
GPU 50% vs. GPU 75%	0.04 [0.03–0.09]	<0.0001
GPU 75% vs. GPU 100%	0.02 [0.01–0.05]	<0.0001
GPU 10% vs. GPU 100%	0.34 [0.25–0.47]	<0.0001

**Figure 3:**
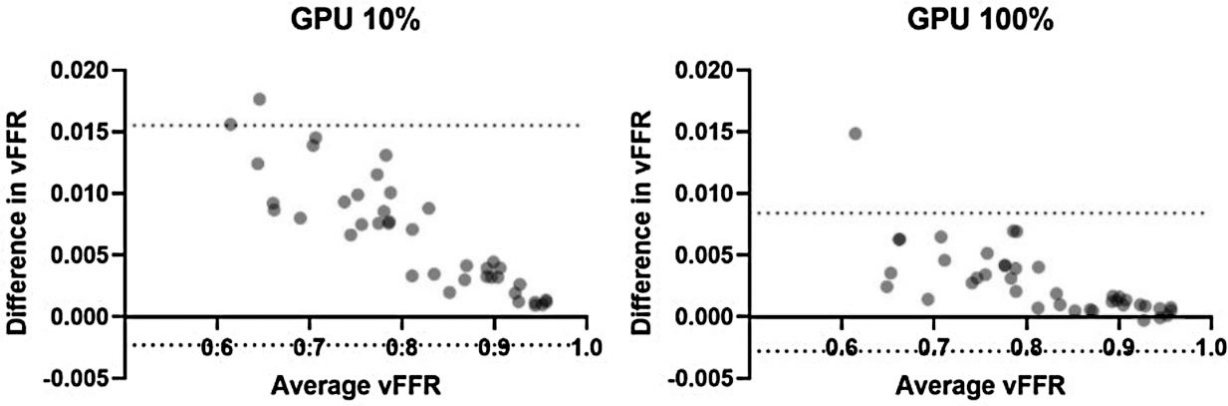
Bland–Altman plots of mean virtual fractional flow reserve (*x* axis) vs. difference in virtual fractional flow reserve between central processing unit and graphics processing unit methods (*y* axis) at 10% fidelity (left) and 100% fidelity (right). Difference was calculated as central processing unit virtual fractional flow reserve minus graphics processing unit virtual fractional flow reserve at a given fidelity. vFFR, virtual fractional flow reserve; CPU, central processing unit; GPU, graphics processing unit.

Accuracy at different fidelities was also explored by Bland–Altman analysis (*Figure 3*). At 10% fidelity, the mean difference in vFFR was +0.0066 [95% limits of agreement (LOA) −0.0023 to 0.0155]. At 100% fidelity, the mean difference in vFFR was reduced to +0.0028 (95% LOA −0.0028 to 0.0084).

**Figure 4:**
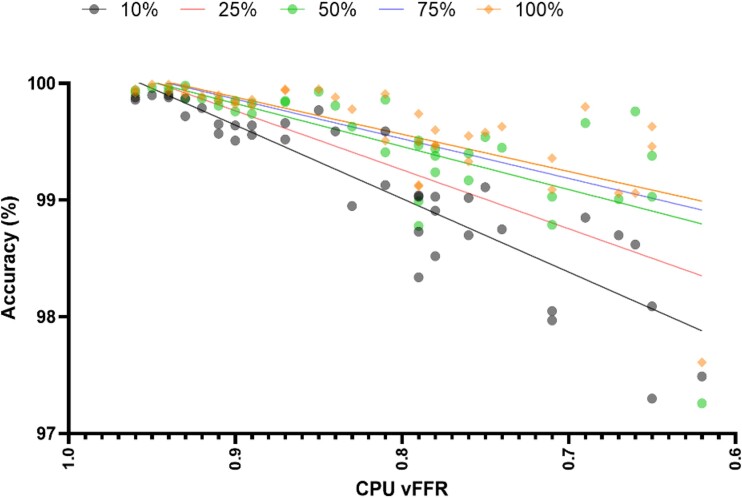
Plot of accuracy of each simulation relative to the central processing unit virtual fractional flow reserve for each graphics processing unit fidelity. Lines represent the simple linear regression model for each graphics processing unit fidelity. Symbols represent individual simulation values for 10, 50 and 100% graphics processing unit fidelity. CPU, central processing unit; vFFR, virtual fractional flow reserve; GPU, graphics processing unit.

The impact of disease severity measured by CPU vFFR upon the accuracy of the GPU method, as assessed at all five fidelities using linear regression models (*Figure 4*), revealed that lower vFFR values were associated with lower accuracy. This relationship was strongest at 10% fidelity (*R*^2^ = 0.81) and weakest at 100% fidelity (*R*^2^ = 0.52) (Prism Internal Slope Comparison *P* < 0.0001). There was no significant change in the relationship between accuracy and vFFR when the GPU fidelity was set at 50% and 100% (*P* = 0.52) and 75% and 100% (*P* = 0.76). At all GPU fidelities, there was 100% concordance between CPU and GPU methods either side of the ≤0.80 vFFR threshold. The GPU method, therefore, never produced a result that would prompt the clinician to perform angioplasty where the CPU method would not, and *vice versa*.

### Comparison of costs and energy consumption

**Figure 5:**
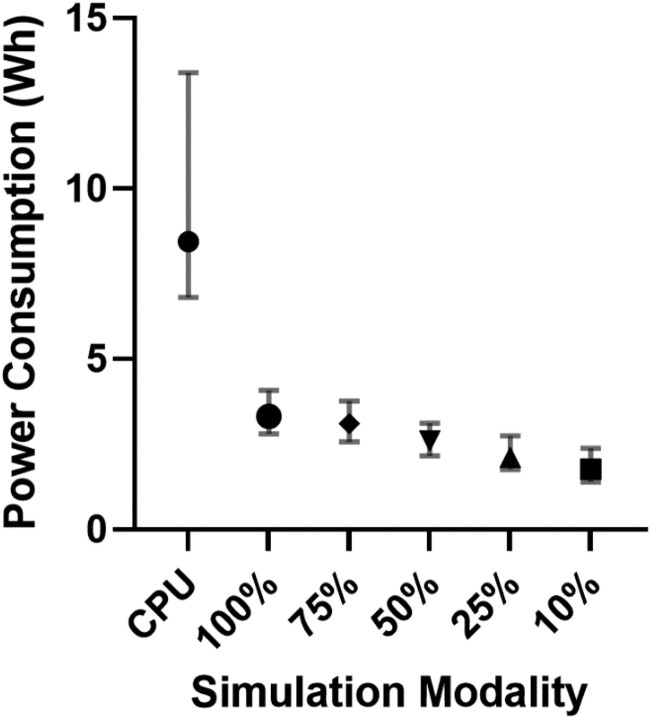
Plot of median +/− IQR power consumed per simulation for different computation methods.

At the time of writing, the CPU used in this study, an Intel Xeon 6136 Gold processing unit, costs £2876. The GPU used, an Nvidia RTX 6000, costs £4080 GBP. The power consumption of each method is shown in (*Figure 5*). The median energy consumption per case using the CPU method was 8.44 (IQR 6.80–13.39) Watt hours (Wh). Significantly less energy was consumed per case by the GPU method when results were pooled across all five fidelities; 2.60 (IQR 2.16–3.12) Wh (*P* < 0.0001). This difference remained significant when the CPU method was compared to the 100% fidelity method [3.32 (IQR 2.81–4.09) Wh (*P* < 0.0001)]. The power consumed at 100% fidelity was significantly greater than that at 10% fidelity [1.77 (IQR 1.40–2.38) Wh (*P* < 0.0001)]. Using an approximate cost of 25 pence per kWh, the median cost of 1000 simulations using the CPU method would be 2110 GBP (8440 kWh) vs. 830 GBP for the GPU method at 100% fidelity (3320 kWh) and 442 GBP at 10% fidelity (1768 kWh).

## Discussion

We have developed and validated a new 3D-CFD method that calculates vFFR in ∼4% of the time taken by the previous fastest method, without a clinically significant reduction in accuracy relative to our current best technique. The novel method is now capable of ‘on-table’ results in ∼30 s, appropriate for live clinical decision making, and requires no additional medications or use of a pressure wire just substitution of software within the clinical application and utilization of a GPU equipped computer. A summary of the computational workflow is show in Supplementary material online, *Appendix Section A.3*.

The convenience, speed, and accuracy of this technique have implications for computing intracoronary physiology. In addition, this technique is more energy efficient than existing methods, improving the sustainability of coronary physiological assessment. This study was not a validation of vFFR accuracy against *in vivo* measurements, which has been studied extensively elsewhere, rather it was an assessment of the speed, accuracy, and cost of a new approach to how vFFR is computed, relative to our established gold-standard method.^6,16–18^ Furthermore, recent work has highlighted additional personalization required to make accurate comparisons between measured and virtual FFR, data that was not available to us in this study.^19^

Existing methods for deriving FFR from angiography with comparable processing times apply simpler mathematical concepts that quantify the inertial and viscous energy losses along a diseased artery, based upon the laws of Bernoulli and Poiseuille. Although this may be sufficient to characterize the more dominant translesional fluid dynamics, their inherent assumptions can neglect important subtleties since they are based on fluid flow in straight conduits, under steady-flow conditions, conditions that are contrary to human coronary anatomy and physiology.^20,21^ Moreover, these simpler laws may predict full pressure recovery distal to a stenosis where the reference arterial diameter is restored. In reality, flow distal to a stenosis becomes disturbed and the disturbed, non-laminar flow patterns exert less pressure on the vessel walls.^7^ Computational fluid dynamics is capable of taking account of this variability relative to the angiographic geometry, improving the accuracy of simulation relative to real life.

This study builds upon existing work and is consistent with results found using other CFD software.^6^ The GPU method uses a well validated and commercially available piece of software used mainly by engineering companies that is well supported and under continual development and improvement. The close match of the GPU and CPU method solutions supports its accuracy and reliability. We used a computer equipped with hardware realistically affordable by most cardiac catheter laboratories. A strength of this study was that the median measured FFR was 0.78 with 75% of the lesions tested having a measured FFR between 0.69 and 0.86. The results of this study therefore reflect accuracy of the GPU method closely around the decision making threshold of FFR ≤0.80. Despite this, not a single case crossed the threshold as a result of GPU computation, and results of the novel method were always concordant with our current gold-standard technique of CPU vFFR measurement. This means that no cases would have received a different treatment (coronary stenting vs. medical therapy) based on the method used to compute vFFR.

Nonetheless, this study has some limitations. The power consumption calculations were based upon assumptions rather than active measurements, although any comparisons tended to favour the existing rather than the novel techniques. For example, it was assumed that the average power consumption of the GPU was its maximum, 295 W. At lower fidelities, however, this was likely to be an overestimate. The GPU method also demonstrated a trend towards lower accuracy at lower vFFRs and a wider spread of accuracies. This is consistent with what is expected with CFD encountering tighter stenoses therefore having to simulate more unpredictable disturbances in flow. The strength of relationship shown by the linear regression at 10% GPU fidelity demonstrated that a simple numerical correction using this equation could be an option to maintain the speed and low power consumption of this method variation. It is also important to note that this was a study of 40 cases, a relatively small number, and further validation *in vivo* is required to confirm these findings.

Future work will address the ability of the method to assess vessels with more than one stenotic lesion during a single simulation. Furthermore, the correction of the 10% fidelity GPU method using the equation derived from linear regression could be explored to test whether this would make any clinically significant improvement in accuracy. Direct assessment of power consumption using the GPU method could also be performed to improve economic and sustainability analyses.

## Conclusions

Graphics processing unit-based methods that use full 3D-CFD can compute vFFR accurately, expediently, and economically with almost identical results to established CPU-based CFD methods. Compared with established methods, the GPU method computed vFFR with up to 28-fold acceleration, yet no significant sacrifice in accuracy. This approach appears apposite for clinical workflows.

## Supplementary Material

ztad028_Supplementary_Data

## Data Availability

All data used for this study are in the manuscript or supplementary material. Requests for additional data will be considered by the corresponding author on reasonable request.
