# Supplementary material for: Rapid virtual fractional flow reserve using 3D computational fluid dynamics
Source: Eur Heart J Digit Health. 2023 Apr 21;4(4):283–90. doi: 10.1093/ehjdh/ztad028 (PMC10393878; doi:10.1093/ehjdh/ztad028)
Supplement: ztad028_Supplementary_Data [file ztad028_supplementary_data.docx]

Supplementary Appendix: VIRTU-rapid

Section A.1

Table 1: Details of cases analysed

| **Case** | **Vessel Type** | **Visual Stenosis** | **CPU vFFR** | **GPU 10%** | **GPU 25%** | **GPU 50%** | **GPU 75%** | **GPU 100%** |
| --- | --- | --- | --- | --- | --- | --- | --- | --- |
| 1 | LAD | 65% | 0.711 | 0.696 | 0.699 | 0.703 | 0.704 | 0.704 |
| 2 | LMS | 70% | 0.952 | 0.952 | 0.952 | 0.952 | 0.952 | 0.951 |
| 3 | LAD | 70% | 0.655 | 0.636 | 0.644 | 0.649 | 0.651 | 0.651 |
| 4 | Cx | 90% | 0.666 | 0.657 | 0.657 | 0.660 | 0.660 | 0.660 |
| 5 | LAD | 65% | 0.694 | 0.685 | 0.690 | 0.692 | 0.693 | 0.693 |
| 6 | Cx | 60% | 0.869 | 0.867 | 0.868 | 0.868 | 0.867 | 0.867 |
| 7 | LAD | 60% | 0.813 | 0.810 | 0.811 | 0.811 | 0.811 | 0.811 |
| 8 | LAD | 60% | 0.757 | 0.748 | 0.751 | 0.752 | 0.753 | 0.753 |
| 9 | OM | 60% | 0.924 | 0.922 | 0.922 | 0.922 | 0.922 | 0.922 |
| 10 | OM | 70% | 0.929 | 0.927 | 0.927 | 0.928 | 0.927 | 0.927 |
| 11 | RCA | 30% | 0.853 | 0.851 | 0.852 | 0.852 | 0.851 | 0.851 |
| 12 | LAD | 50% | 0.927 | 0.927 | 0.927 | 0.926 | 0.926 | 0.926 |
| 13 | LAD | 60% | 0.779 | 0.768 | 0.771 | 0.773 | 0.774 | 0.775 |
| 14 | LAD | 50% | 0.905 | 0.903 | 0.904 | 0.904 | 0.903 | 0.903 |
| 15 | RCA | 90% | 0.622 | 0.610 | 0.605 | 0.604 | 0.603 | 0.601 |
| 16 | RCA | 70% | 0.665 | 0.655 | 0.656 | 0.664 | 0.660 | 0.660 |
| 17 | LAD | 60% | 0.793 | 0.782 | 0.783 | 0.785 | 0.786 | 0.787 |
| 18 | RCA | 65% | 0.872 | 0.869 | 0.870 | 0.870 | 0.870 | 0.869 |
| 19 | Cx | 75% | 0.790 | 0.783 | 0.785 | 0.786 | 0.785 | 0.786 |
| 20 | LAD | 80% | 0.650 | 0.637 | 0.642 | 0.646 | 0.647 | 0.648 |
| 21 | LAD | 30% | 0.789 | 0.775 | 0.777 | 0.780 | 0.782 | 0.783 |
| 22 | RCA | 90% | 0.815 | 0.808 | 0.809 | 0.810 | 0.811 | 0.812 |
| 23 | LAD | 30% | 0.945 | 0.944 | 0.944 | 0.944 | 0.944 | 0.944 |
| 24 | LAD | 65% | 0.760 | 0.752 | 0.753 | 0.754 | 0.754 | 0.755 |
| 25 | LAD | 40% | 0.945 | 0.944 | 0.944 | 0.944 | 0.944 | 0.944 |
| 26 | RCA | 50% | 0.837 | 0.834 | 0.835 | 0.835 | 0.835 | 0.834 |
| 27 | Cx | 50% | 0.742 | 0.733 | 0.736 | 0.738 | 0.739 | 0.739 |
| 28 | LAD | 50% | 0.748 | 0.741 | 0.743 | 0.744 | 0.745 | 0.745 |
| 29 | LAD | 50% | 0.908 | 0.905 | 0.906 | 0.906 | 0.906 | 0.906 |
| 30 | Cx | 50% | 0.899 | 0.897 | 0.897 | 0.896 | 0.896 | 0.896 |
| 31 | LAD | 35% | 0.833 | 0.826 | 0.829 | 0.830 | 0.830 | 0.830 |
| 32 | RCA | Missing | 0.957 | 0.956 | 0.956 | 0.956 | 0.956 | 0.956 |
| 33 | Cx | 60% | 0.957 | 0.956 | 0.956 | 0.956 | 0.956 | 0.955 |
| 34 | LAD | Missing | 0.785 | 0.777 | 0.779 | 0.780 | 0.780 | 0.781 |
| 35 | LAD | 40% | 0.894 | 0.891 | 0.891 | 0.891 | 0.891 | 0.892 |
| 36 | LAD | 70% | 0.893 | 0.891 | 0.892 | 0.892 | 0.892 | 0.891 |
| 37 | LAD | 80% | 0.778 | 0.771 | 0.772 | 0.774 | 0.774 | 0.775 |
| 38 | LAD | 70% | 0.714 | 0.699 | 0.704 | 0.707 | 0.708 | 0.709 |
| 39 | RCA | 80% | 0.789 | 0.782 | 0.784 | 0.786 | 0.786 | 0.787 |
| 40 | Cx | 80% | 0.901 | 0.897 | 0.898 | 0.899 | 0.899 | 0.899 |

Section A.2: Detailed Results Comparison

Purpose

To demonstrate the differences of the vFFR results throughout the vessel between the GPU method and CPU method at two fidelities. Even though, in practice, only the vFFR computed based on the pressure drop at the outlet and the inlet matters, we compute it here to help you understand why the results varied.

Methodology

Four cases that span the vFFR prediction differences range—high, mid, mid-low, and low difference—were selected for this illustration.

Two configurations were tested in each of these cases with 10% and 100% fidelities. Each with a flow rate of 1 ml/s and 3 ml/s. At several sample points located at the vessel's centre and evenly distributed along its length from the inlet to the outlet, the resulting pressures were measured. To calculate the vFFR, pressure drops between the inlet and the sample locations were computed. The difference between the vFFR and the vFFR calculated using the CPU approach was measured.

Observations

Results for the 4 cases have been plotted in Figures 1A, 1B, 1C, and 1D.

For all the cases it can be observed as one moves from the inlet towards the outlet the difference in the results between the GPU method and CPU method grows. The location where the spike in the difference is observed is near and after the stenosis. This is expected in CFD, as the flow is most complex there and minor differences in numerical modelling can affect results. This difference is then carried downstream towards the outlet. Despite these variations, the vFFR measurements remain within allowable practical variations.

Figure 1A Figure 1B

 Figure 1C Figure 1D
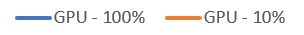


Section A.3: Workflow

Figure 2

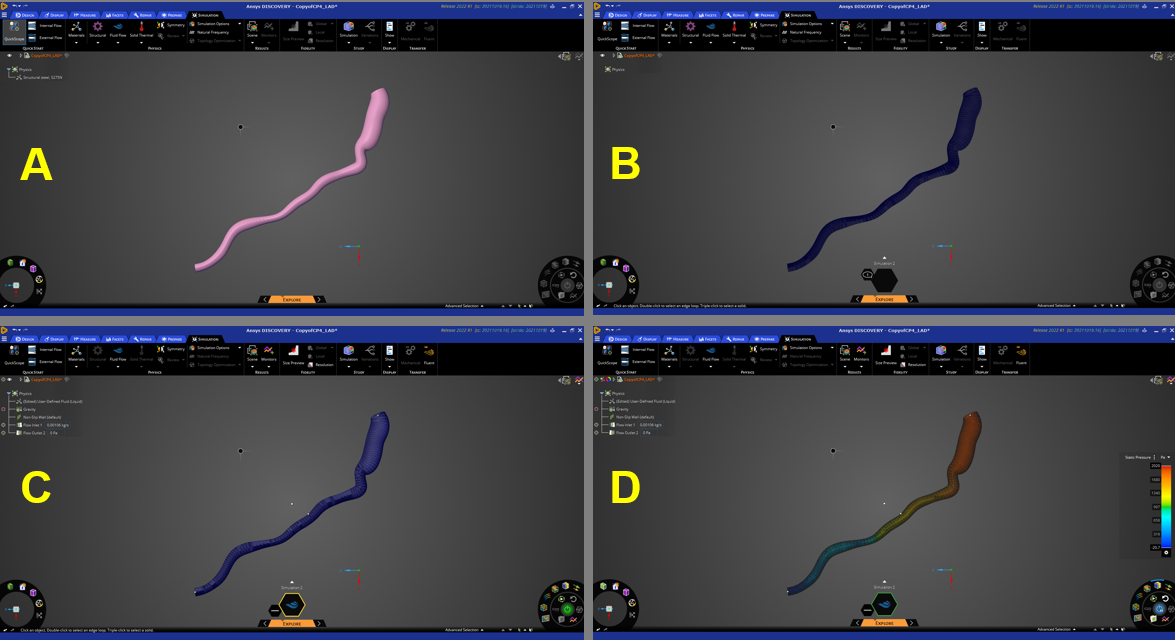


Figure 2. The novel GPU-based workflow. Screenshots from ANSYS Discovery show a reconstructed, diseased left anterior descending artery being analysed. The surface geometry is loaded (A) which is converted from a surface into a volume (B). The simulation is then set up (C) and the results with colour mapped pressure distribution is shown (D). The steps to convert the angiogram images into a surface volume are described previously. (6,11)
